# Topics of the Month

**Published:** 1894-01

**Authors:** 


### TOPICS OF THE MONTH.

A SPECIAL committee appointed by the President of the Medical Society of the County of New York in April last, to consider the propriety of proposing an amendment to the medical laws regulating the practice of obstetrics by midwives in the State of New York, has recently made its report. The proposed bill consists of ten sections, and may be summarized as follows: First, every person practising midwifery shall possess a certificate from the State Board of Medical Examiners. Second, within thirty days after the passage of the act, every such person shall apply to the State Board of Medical Examiners, under oath, for a certificate authorizing the person named therein to practise midwifery in this State. Third, every person hereafter beginning the practice of midwifery shall be examined by the State Board, and, if successful, shall be entitled to a certificate. Fourth, certificates are to be filed in the county clerk's office. Fifth, licenses may be refused to persons of bad character, or who are guilty of unprofessional or dishonest conduct, and the State Board may revoke licenses for similar causes. Sixth, fees and penalties are prescribed, and the usual details with reference to explanation of the meaning of the act are given. We believe this is a beneficial and humane proposition, and it should be passed by the Legislature without opposition. A statute regulating the practice of midwifery uniformly throughout the State has long been needed.

---

THE manager of the Daggett Table Company, who was recently arrested at the instance of Mr. Anthony Comstock, special inspector of the Post Office Department, for the alleged transmission of obscene literature through the mails, has been discharged by Commissioner Fairchild, on the *ex parte* statement of Mr. Comstock himself. It appears that the Daggett Table Company has issued an illustrated catalogue, with male and female figures posed in various postures on the several tables that the company manufacture, and have sent these pamphlets to the profession of medicine through the mails, which is the head and front of the offending to Mr. Comstock's over-sensitive occular apparatus.

The tables in question were invented by Dr. B. H. Daggett, of Buffalo, a physician in regular practice, and in good standing in the several medical societies—city, county and State—of which he is a member. He is, moreover, a surgeon of skill, and has displayed much originality in devising many mechanical appliances used in the surgical art. At first, he manufactured the tables himself, but as the business increased in its proportions a company was organized, and Mr. B. B. Daggett, son of the inventor, was appointed its manager. This young man was arrested, as has already been stated, the business was brought to a standstill, and so held for about two months, but he was finally discharged without being obliged to offer any defense, the prosecution having failed to make a case. Such a mistaken interpretation of the law as Mr. Comstock made, and the gross injustice perpetrated on individual rights, ought not to pass without the severe condemnation of every good citizen.

---

The Maltine Manufacturing Company, of New York, has issued an attractive calendar for 1894, consisting of six folios, with two months recorded on each folio. Each page has also something to say with reference to maltine, and its various combinations, used in the treatment of disease.

But more than this: each page contains a photogravure of a prominent physician, the following names comprising the list: Dr. Joseph D. Bryant, of New York, Dr. William Pepper, of Philadelphia, Dr. N. S. Davis, of Chicago, Dr. Henry O. Marcy, of Boston, Dr. Hunter McGuire, of Richmond, and Dr. Lewis A. Sayre, of New York. With the exception of Dr. Pepper, it will be observed that these are all conspicuous examplars of the code of ethics of the American Medical Association. They have been conspicuously prominent of late in waiving the banner of the code over the American medical profession, and have especially denounced advertising by the publication of interviews or personal pictures in the newspaper press. What shall be said of men who preach one doctrine for the mass of the profession of medicine, and practise another for themselves? For, assuredly, there can be little ethical difference between permitting the publication of photographs in newspapers and in a neat advertising medium like the one in question.

---

The recent death of Dr. Edward Warren-Bey calls to mind many circumstances connected with his picturesque career. The journals have chronicled the details of his early life and his noted professional career in America, including his service as surgeon in the Confederate army, and his return to Baltimore after the war, where he resumed his medical practice and became professor of surgery in the Washington University Medical School. It was during this time that Dr. Warren became a conspicuous figure in the famous Wharton–Ketchum murder trial in 1871. It is related that Dr. Warren, in the course of this trial, made a retort to a lawyer that rendered him famous throughout the world. The Attorney-General of Maryland, Mr. Syester, who was conducting the case for the prosecution, having pressed Dr. Warren sharply for answers favorable to his cause, in which he was foiled, the

Attorney-General exasperatingly exclaimed, "Doctor's mistakes are generally buried." "Yes," was Dr. Warren's quick reply, "and lawyer's mistakes are frequently hung."

---

THE fog-horn epidemic has finally reached Buffalo, and, to the almost endless catalogue of noise nuisances already existing, must be added that of the fog-horn nuisance. For the past three months and more, night and day, the people of Buffalo have been reminded of the fact that this prosperous city is also one of the most important ports on the great lakes. Nerves have been jarred in the day time and sleep has been disturbed at night by the almost never-ceasing tooting of this abomination of modern civilization. While it must be confessed that it is humane and proper to take timely and abundant care to prevent disaster to the life and property of navigators of the lakes, it must be admitted that those who consent by force of circumstances to reside upon land have some rights that ought to be respected. Without entering into a detailed argument at this time as to reasons pro and con on this subject, we shall content ourselves with merely remarking that if we must have fog horns it would be eminently proper to place them sufficiently out to sea to prevent their intolerable tootings from destroying the health and comfort of people who reside on land.

---

WE WROTE cheerfully last month of the deliverance of the people of Buffalo from the discomforts of travel in the cold street cars, basing our statement upon a published interview with Manager Littell, who, it was alleged, had stated that stoves had arrived and would be placed in all the cars as soon as the work could be conveniently and properly done. But it seems that we were too sweeping in our assertions. The good people residing along the Elmwood avenue route and its contributory streets, according to Mr. Littell's latest edict, are not to enjoy the luxury of warm street cars this Winter. We are thankful, however, for even some relief, and perhaps in due time the entire city will be adequately and comfortably served with cleanly and well warmed street cars.

---

DR. HERMANN M. BRIGGS, bacteriologist to the Health Department of New York, has recently made a report, in which he shows that more than 6,000 deaths were reported in that city last year as due to consumption. It will thus readily be understood that this is the most common and fatal disease prevailing in New York. What is true of New York, in this regard, is likewise true of the country in general, excepting, of course, some specially favored localities, wherein the disease is comparatively unknown. It is high time that people were awakened to a knowledge of the fact that consumption is not only a communicable disease, but also that it is absolutely preventable when all known factors of prevention are invoked.

It is a well-known fact that dried sputum, broken up into fine particles, is one of the most highly dangerous methods of communicability. It is terrible to contemplate the carelessness with which consumptives are permitted to travel by rail and water.

Medical Director Albert L. Gihon, U. S. N., in his address as president of the section of Hygiene, Climatology and Demography, in the first Pan-American Congress, used the following words:

The consumptive, whose traits no professional acumen is required to recognize, frequents our crowded thoroughfares, sits beside us in unventilated street cars and at the hotel table, occupies Pullman sleeping berths, and shares the steamship state-room, wholly unrestrained and innocently ignorant that he or she may be sowing the seeds of disease among delicate women and children. Any one may verify this who uses his eyes for the purpose along the railway and coastwise steamer routes to our invalid resorts. Within a twelvemonth, on my way to Mexico by rail, I was a fellow-passenger with two invalids in the advanced stage of phthisis, en route for San Antonio, one of whom occupied the opposite berth, and the other one diagonally across the car, so that I could see and hear them coughing and expectorating, with only such attention as well-intending but unskilled relatives could render. They had no vessels for receiving their sputa, which was discharged in their pocket handkerchiefs, to be scattered over pillows, coverlets and blankets. They left the car in the morning, and I saw those same berths, it is true with change of linen sheets and pillow-cases, but with no change of blankets, mattresses, or pillows, occupied that very night by other travelers, who were thus subjected to contact with a pathogenic microbe far more tenacious of life and power of evil-doing than the dreaded cholera spirillum.

One has only to sit in a crowded street car on a winter day, and watch the clouds of respiratory steam circling from the mouths and nostrils of the unclean and diseased into the mouths and nostrils of the clean and healthy, as the expiratory effort of the one corresponds with the inspiratory act of the other. The road is short, but straight and sure, from vomica and mucous patch to the receptive nidus in another's body. Who that has ever had forced upon him an aerial feast of cabbage, onions, garlic, alcohol, tobacco, and the gastric effluvia of an old débauché, can doubt that aqueous vapor can transport microscopic germs by the same route? Not long ago I traveled by sea from New York to Charleston, and for two nights was cabined with some twenty consumptives going to Florida. The air was chill, and they huddled around the stoves and fearfully and fearlessly closed doors and windows, until the atmosphere became stifling with their emanations and the dried sputa, which they ejected on every side. It was comparatively easy to escape during the day by staying on deck, and I slept with my state-room windows wide open, but the curtains, carpets, pillows, and mattresses had been saturated by I know not how many expectorating predecessors. I have visited fifty small-pox patients a day, have gone through yellow fever wards, and stood by cholera bedsides with far less apprehension than I experienced on that trip, yet it was one taken by many thousands of people, who would have been terrified to know that a case of cholera was within a mile to leeward of their homes.

Here is a picture that is a sad one to contemplate, yet it is a fact that all railway travelers, and even, to a certain extent, those who ride in steamships, are exposed to similar dangers, while innocently regarding themselves in an atmosphere of safety. It is high time that the people became aroused to the necessity of correcting evils that are fraught with such subtle and terrible danger.

---

General George S. Field, President of the Board of Public Works of Buffalo, if he is correctly reported, has recently said that three-storied school houses have already been decided on by the commissioners, since it has been found that children do not object to going up stairs and the doctors even recommend it, stating that the exercise brought new sets of muscles into play. General Field is admittedly an excellent engineer and withal a famous traveler, but he evidently falls in the rear of the column of progress when he discusses the school house question from the standpoint of the foregoing interview.

We are at a loss to know how any doctor of standing in his profession could have advised in the manner quoted by General Field. It can only be accounted for on the hypothesis that the doctors consulted know more of politics than of school house sanitation. That the children do not object to going up stairs is no argument in favor of the plan, since they are, for the most part, too young to exercise intelligent judgment on the subject. While it is true that the exercise of climbing stairs brings into play muscles that are little used on a level, yet it is also quite true that this slight advantage is counterbalanced manyfold by the disadvantages which result from hastily climbing flights of stairs by school children. This is especially true of girls between the ages of ten and sixteen, when their pelvic organs are developing and are otherwise undergoing changes to fit them for useful womanhood. The mere act of going up and down stairs is not deleterious in and of itself, provided the transit be performed slowly and the flights are few and short; but who ever knew of a group of school children to go up and down stairs slowly? No child, however, can go up even two flights consecutively without becoming unbreathed. We will not quarrel with the number of stories that General Field puts upon his school houses, provided he makes plans for keeping the girls on the ground floor. But, since this is impossible in accordance with the present graded school system, we advise General Field to countermand all orders for school houses over two stories high.

---

A NEW and elegant operating room was opened at the Sisters of Charity Hospital last month, which, for convenience and adaptability, is unsurpassed. It consists of a central amphitheater surrounded by four separate rooms, one for dressing, one for the surgeon, and two for narcotizing patients. The amphitheater has an inlaid cement floor slanting toward the center, where it opens, properly trapped, into the sewer. The side walls are of tile, and the ceiling of heavily enameled plaster; three rows of seats, about 100 in number, surround the operating table in such a manner that the view is unobstructed.

Altogether, it presents a very neat and business-like appearance, and does great credit to the sisters in charge, proving that they are wide-awake and appreciate the enormous demands and possibilities of modern antiseptic surgery. We take pleasure in congratulating them and our city on the possession of such a gem, and predict that the public will not be slow in availing itself of it when in need. The clinics of the medical department of Niagara University will be held there every Wednesday and Saturday from 10 to 12 A. M., and the profession is cordially invited by Dr. Mynter to be present.

---

THE church-bell nuisance continues to menace the health and life of the community, notwithstanding the numerous requests that have been made from time to time for its suppression. If there can be anything more exasperating or trying to the nerves than for a sick person to be awakened at four or five o'clock in the morning by the numerous bells in St. Michael's tower, miscalled chimes, we do not know it. It is high time that the health authorities interfered in this matter and suppressed this menacing and almost intolerable nuisance.

---

The County Hospital plan, by which it is proposed to convert the Erie County Almshouse into a hospital, with a staff of attending physicians and surgeons, is making progress. The differences heretofore existing among those interested in the scheme seem to have been adjusted, and it now only remains for the Board of Supervisors to do their duty to consummate this humane and greatly needed change. We hope to be able to chronicle next month the details of the plan and to publish the names of the staff that have been agreed upon.

